# Multimodal 7T Imaging of Thalamic Nuclei for Preclinical Deep Brain Stimulation Applications

**DOI:** 10.3389/fnins.2016.00264

**Published:** 2016-06-10

**Authors:** YiZi Xiao, Laura M. Zitella, Yuval Duchin, Benjamin A. Teplitzky, Daniel Kastl, Gregor Adriany, Essa Yacoub, Noam Harel, Matthew D. Johnson

**Affiliations:** ^1^Department of Biomedical Engineering, University of MinnesotaMinneapolis, MN, USA; ^2^Center for Magnetic Resonance Research, University of MinnesotaMinneapolis, MN, USA; ^3^Institute for Translational Neuroscience, University of MinnesotaMinneapolis, MN, USA

**Keywords:** thalamus, high-field imaging, susceptibility weighted imaging, diffusion weighted imaging, fiber tractography

## Abstract

Precise neurosurgical targeting of electrode arrays within the brain is essential to the successful treatment of a range of brain disorders with deep brain stimulation (DBS) therapy. Here, we describe a set of computational tools to generate *in vivo*, subject-specific atlases of individual thalamic nuclei thus improving the ability to visualize thalamic targets for preclinical DBS applications on a subject-specific basis. A sequential nonlinear atlas warping technique and a Bayesian estimation technique for probabilistic crossing fiber tractography were applied to high field (7T) susceptibility-weighted and diffusion-weighted imaging, respectively, in seven rhesus macaques. Image contrast, including contrast within thalamus from the susceptibility-weighted images, informed the atlas warping process and guided the seed point placement for fiber tractography. The susceptibility-weighted imaging resulted in relative hyperintensity of the intralaminar nuclei and relative hypointensity in the medial dorsal nucleus, pulvinar, and the medial/ventral border of the ventral posterior nuclei, providing context to demarcate borders of the ventral nuclei of thalamus, which are often targeted for DBS applications. Additionally, ascending fiber tractography of the medial lemniscus, superior cerebellar peduncle, and pallidofugal pathways into thalamus provided structural demarcation of the ventral nuclei of thalamus. The thalamic substructure boundaries were validated through *in vivo* electrophysiological recordings and post-mortem blockface tissue sectioning. Together, these imaging tools for visualizing and segmenting thalamus have the potential to improve the neurosurgical targeting of DBS implants and enhance the selection of stimulation settings through more accurate computational models of DBS.

## Introduction

Structural brain imaging has become a valuable tool to guide the implantation and programming of deep brain stimulation (DBS) systems for the treatment of numerous brain disorders (Butson et al., 2007; Lemaire et al., 2007; Larson et al., 2012b). Current clinical magnetic resonance imaging (1.5-3T) provides reasonable imaging contrast to identify, for example, the borders of the globus pallidus and to some extent the borders of the subthalamic nucleus (Starr et al., 1999) for treatment of Parkinson's disease. Such visualization abilities have enabled new opportunities for interventional MRI guided stereotactic neurosurgery (Larson et al., 2012a).

However, clearly demarcating targets within the thalamus, another surgical target of DBS, at these field strengths remains a considerable challenge for both clinical (Schlaier et al., 2005; Abosch et al., 2010) and preclinical DBS studies. Improvement in structural imaging of intra-thalamic nuclei would have important implications given that interventional stereotactic procedures within thalamus have shown marked promise for the treatment of pain (Levy et al., 1987), essential tremor (Benabid et al., 1991; Lipsman et al., 2013), epilepsy (Takase et al., 2009; Fisher et al., 2010), Tourette syndrome (Visser-Vandewalle et al., 2003), disorders of consciousness (Schiff et al., 2007), with other brain disorder indications on the horizon including schizophrenia (Ewing et al., 2013; Klein et al., 2013). This is especially important because favorable behavioral outcomes with thalamic DBS hinge upon the accuracy of stimulating the desired thalamic pathway, while avoiding modulation of neuronal pathways implicated in the emergence of adverse side effects (Akbostanci et al., 1999; Papavassiliou et al., 2004; Kuncel et al., 2008; Yu et al., 2009; Keane et al., 2012).

Thalamic nuclei can be difficult to visualize with traditional (1.5-3T) scanners, thus requiring the identification of fixed coordinates based on an internal reference, such as the anterior commissure and posterior commissure plane (Schaltenbrand and Wahren, 1977; Villemure et al., 1987; Lunsford, 1988; Talairach and Tournoux, 1988; Kall et al., 1992). However, several imaging approaches have been used to demarcate various thalamic nuclei beyond typical clinical imaging protocols. These include functional imaging (Baker et al., 1996; Samuel et al., 1997; Davis et al., 1998; Krams et al., 1998), high field magnetic resonance imaging (Calamante et al., 2012, 2013; Lenglet et al., 2012; Tourdias et al., 2014), parcellation utilizing corticothalamic diffusion-weighted imaging with probabilistic tractography (Behrens et al., 2003; Johansen-Berg et al., 2005), and other signal processing techniques (Deoni et al., 2005; Gringel et al., 2009).

Another approach to capture subtle thalamic anatomy is using histologically derived brain atlases (Gee et al., 1991) based on acetylocholinesterase (AChE) (Mesulam et al., 1984) and calcium-binding protein (e.g., parvalbumin) (Lanciego and Vazquez, 2012) labeling of thalamus (Morel et al., 1997) to match and overlay upon individual MR images (Deoni et al., 2005; Gringel et al., 2009). However, inter-subject variability in thalamic anatomy has been widely demonstrated (Galaburda et al., 1978; Eidelberg and Galaburda, 1982; Good et al., 2001), and a “one size fits all” method for linear registration of histological brain atlases to structural imaging data has proven to be imprecise amongst subjects (Starr et al., 1999). The need to identify anatomical information within individual MR imaging data has prompted the development of deformable digital atlases (Bertrand et al., 1973; Bohm et al., 1983; Dann et al., 1988; Evans et al., 1988; Marrett et al., 1989; Greitz et al., 1991; St-Jean et al., 1998; Nowinski et al., 2000; Finnis et al., 2003; Ganser et al., 2004; Chakravarty et al., 2006; Yelnik et al., 2007; Dauguet et al., 2009; Sadikot et al., 2011), numerous image processing techniques (Mcinerney and Terzopoulos, 1996; Maintz and Viergever, 1998; Wolberg, 1998; Pham et al., 2000; Pitiot et al., 2006), and intraoperative microelectrode mapping procedures to verify and expand upon the interpretation of the imaging data (Hamani et al., 2005; Gross et al., 2006).

**Figure 1:**
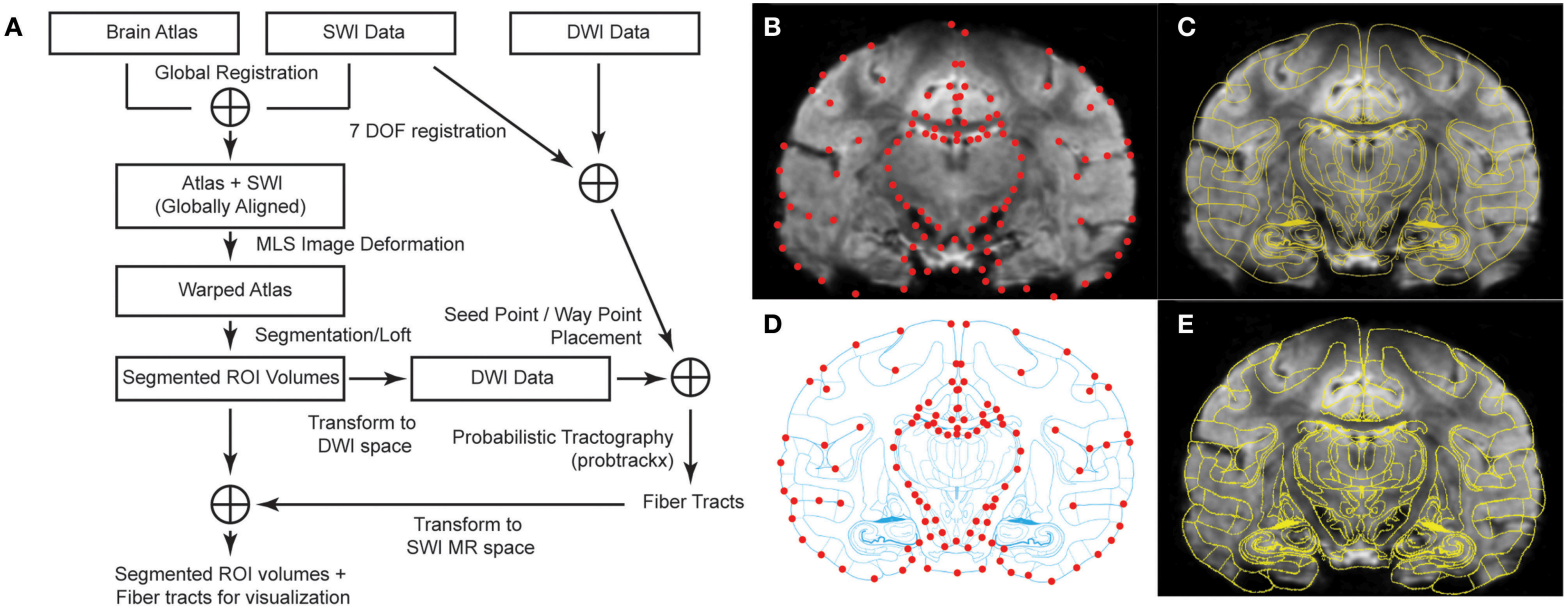
**Brain atlas registration and warping process to SWI from Subject 3. (A)** Schematic of the methodological analysis process combining SWI and DWI data. **(B)** Coronal image with 94 manually selected control points in red (q points). **(C)** Overlay of MRI with the corresponding atlas plate after global alignment. **(D)** Atlas plate after global alignment with MRI. Matching control points in red (p points). **(E)** Warped atlas plate superimposed on top of the original MR image.

Here, we show in seven non-human primates that (1) high-field (7T) susceptibility-weighted imaging and high-angular resolution diffusion imaging enable demarcation of intra-thalamic nuclei and (2) high-field images coupled with an intuitive 2D image registration and deformation method using moving least squares (MLS) (Schaefer et al., 2006) provides enhanced visualization of individual thalamic nuclei in three-dimensions (Figure 1A). These methods were further validated through *in vivo* electrophysiological mapping and *ex vivo* blockface tissue sectioning.

## Materials and methods

### Image data acquisition

High-field magnetic resonance imaging (7T, Magnex Scientific) was performed on seven rhesus macaque primates (*macaca mulatta*, 6 female and 1 male, Table 1) at the University of Minnesota's Center for Magnetic Resonance Research using a Siemens console and head gradient insert capable of 80 mT/m with a slew rate of 333 mT/m/s. A customized head coil was developed with 16-channel transmit and 16+6 receive channels, in which 4 coils mounted on top of each subject's head and 2 ear-loop coils were added to enhance signal detection from subcortical structures (Adriany et al., 2010). All procedures were approved by the Institutional Animal Care and Use Committee of the University of Minnesota and complied with United States Public Health Service policy on the humane care and use of laboratory animals. Animals were anesthetized (isoflurane, 2.5%) during the imaging sessions and monitored continuously for depth of anesthesia. Animals were individually housed in a Primate Products Enhanced Environment Housing System (dark/light cycle of 12/12) in the University of Minnesota's Research Animal Resources facility. The animals were given a range of environmental enrichment (e.g., toys, foraging baskets, mirrors, TV), provided with water *ad libitum*, and given a range of food options including fresh fruit and vegetables. All efforts were made to provide good care and alleviate unnecessary discomfort, and no adverse events occurred. At the conclusion of the study and in order to validate the magnetic resonance imaging data, two animals were deeply anesthetized with sodium pentobarbital and perfused with phosphate buffered saline followed by a 4% paraformaldehyde fixative solution, consistent with the recommendations of the Panel on Euthanasia of the American Veterinary Medical Association.

**Table 1:** **Subject characteristics and imaging protocols**.

**Subject**	**Gender**	**Age**	**SWI Resolution**	**DWI**	**EM**	**BFS**	**Description**
1	F	22	0.4 mm iso				
2	F	22	0.4 mm iso				
3	F	14	0.33 mm iso				
4	F	13	0.4 mm iso	≪	≪		EM validation of SWI and DWI
5	F	10	0.4 mm iso	≪		≪	BFS validation of SWI and DWI
6	F	9	0.4 mm iso	≪		≪	BFS validation of SWI and DWI
7	M	4	0.33 mm iso				

(iso: isometric, EM: electrophysiological mapping, BFS: blockface sectioning).

Susceptibility-weighed imaging (SWI) was collected in all subjects (*n* = 7) and consisted of a 3D flow-compensated gradient echo sequence using a FOV of 128 × 96 × 48 mm^3^, matrix size of 384 × 288 × 144 (0.3–0.4 mm isotropic resolution), TR/TE of 35/29 msec, flip angle of 15°, BW of 120 Hz/pixel, and acceleration factor of 2 (GRAPPA) along the phase-encoding direction. SWI is sensitive to a difference in magnetic susceptibility in tissues and can be used to measure iron content (Haacke et al., 2009b), in the form of ferritin and hemosiderin, found in oligodendrocytes (Francois et al., 1981; Dwork et al., 1988; Levine and Macklin, 1990; Schenck and Zimmerman, 2004) and regions of the basal ganglia and thalamus (Haacke et al., 2009a). In this case, a local difference in iron concentration manifests in a difference in local magnetic susceptibility, causing a deviation in the induced magnetization, translating into a difference in phase (Haacke et al., 2009b). Studies have shown that the phase shift is linearly correlated with iron concentration (Ogg et al., 1999; Hopp et al., 2010). Here, we used a T2*-weighted gradient echo sequence and combined the magnitude and phase information by multiplying a “phase mask” to the magnitude image. Values in the phase image above zero were assigned to 1 in the phase mask (i.e., negated), while those between 0 and –π were linearly scaled from 1 to 0. The phase mask was then raised to a power of 4 and multiplied to the magnitude image. The choice of raising the phase mask to the power of 4 was based on optimizing the contrast-to-noise ratio of the SWI image (Haacke et al., 2009b). In this way, regions in the magnitude image with large phase shifts had their magnitudes severely attenuated and appeared hypointense in the SWI data (Haacke et al., 2009b). Whole-brain SWI scans required ~30 min per animal.

Additionally, diffusion-weighted imaging (DWI) was collected and analyzed in a subset of subjects (*n* = 3). DWI consisted of a single refocused 2D single-shot spin echo EPI sequence (Stejskal and Tanner, 1965) using a FOV of 128 × 84 × 99 mm^3^, matrix size of 128 × 84 × 50 (1 mm isotropic resolution), TR/TE of 3500/53 msec, flip angle of 90°, BW of 1860 Hz/pixel, and an acceleration factor of 3 (GRAPPA). Diffusion-weighted images (*b* = 1500 s/mm^2^) were collected with diffusion gradients applied along 55–143 uniformly distributed directions (Deriche et al., 2009). Fifteen additional non-diffusion-weighted images (*b* = 0 s/mm^2^) were acquired for every 10 diffusion-weighted images. We utilized TOPUP (Andersson et al., 2003) in FSL to correct for geometric distortions in the EP images due to magnetic field inhomogeneities. This approach used multiple non-diffusion-weighted (b0) scans with bidirectional (posterior-anterior and anterior-posterior) phase-encoding directions to calculate and counteract the deformation field. Whole-brain DWI scans required ~30 min per animal.

### Atlas registration

#### Global atlas registration

To assist with identification of thalamic nuclei, a rhesus macaque brain atlas (Paxinos et al., 2000) was registered and nonlinearly deformed to the MRI volumes of each of the seven subjects. In preparation, MRI volumes were aligned in AC-PC space (Analyze 11.0, AnalyzeDirect) and then resliced into serial coronal images. A set of 40 coronal images, spanning the entire thalamic region, was extracted from each subject's imaging data set. First, a non-uniform rational B-spline modeling program (Rhinoceros) was used to create a proportional grid system, as developed by Talairach (Talairach and Tournoux, 1988) for the human brain, to identify equivalent slices between the MRI and brain atlas (*n* = 30 slices) (Paxinos et al., 2000). The distance (variable over 7 subjects, average: 0.482 mm, minimum: 0.429 mm, maximum: 0.517 mm) between each slice was then used to generate 10 further images posterior to the PC. An initial global registration of the interpolated MR images to the brain atlas (Paxinos et al., 2000) was performed using both a global rigid transformation and a local affine transformation (Sorlie et al., 1997), such that the cortical outlines and the inter-hemispheric fissures in the atlas section were aligned with those on the MR image (Figures 1B,C).

#### Refined atlas warping

To further warp the atlas to individual MR images, control points p and q were manually placed on each atlas section and corresponding MR image, respectively, such that p_*i*_ and q_*i*_ represent the same spatial location. Common locations for control points were located on the boundaries of the cortex, major sulci, lateral and third ventricles, interpeduncular cistern, and the borders of thalamus (Sorlie et al., 1997; Castro et al., 2006). Spatial selection of control points across MR images with thalamus was consistent with those control points shown in Figures 1B,D. We then used a nonlinear atlas warping approach that adapted a MLS image deformation algorithm (Schaefer et al., 2006). For each pixel *v* in the undeformed image, the algorithm solved for the best transformation function *f*(*x*) that satisfied:

- *f*(*p*_*i*_) = *q*_*i*_
- *f* produces a smooth deformation
- if *p*_*i*_ = *q*_*i*_ → *f*(*v*) = *v*

and minimized

$$\underset{i=0}{\sum^{n}}w_{i}{\left|f\left(p_{i}\right)-\;q_{i}\right|}^{2},$$

where $w_{i}=\;\frac{1}{{\left|p^{i}-v\right|}^{2\alpha }}$ (α = 2 was found to be suitable in this case).

In other words, the handles *p*_*i*_ should map directly to *q*_*i*_ under deformation, and if the deformed handles *q*_*i*_ are the same as *p*_*i*_, then *f* should be the identity function. Since the weights *w*_*i*_ were dependent on the location of each pixel, the algorithm solved for a different *f*(*x*) for each pixel. *f*(*x*) in the most general case was an affine function of the form: *f*(*x*) = *xM*+*T*, where *M* and *T* were rotation and translation matrices, respectively. The affine transformation allowed for rotation, translation, anisotropic scaling, and anisotropic shearing in two-dimensions. For more conservative *similarity* and *rigid* deformations, restrictions were put on the rotational matrix *M* to ensure isotropic shearing and scaling. Closed-form solutions were derived for all three cases.

In cases of large deformations, the sign of the Jacobian of *f*(*x*) can change and the one to one mapping of pixels may be violated, causing the image to fold back on itself. To eliminate such fold-backs, we implemented an approach by Tiddeman et al. to break up the entire warp into a series of smaller partial deformations, ensuring in each step the Jacobian of *f*(*x*) does not change sign (Tiddeman et al., 2001). In each stage, the partially warped image serves as the starting point for a new round of deformation until all the control point restraints *f*(*p*_*i*_) = *q*_*i*_ are satisfied (Figures 1D,E).

### Diffusion tensor imaging

Fiber tractography was performed in FSL (Smith et al., 2004; Woolrich et al., 2009; Jenkinson et al., 2012) for three subjects (M4, M5, and M7) to extract several fiber tract pathways projecting into thalamus. SWI images were converted into NIfTI files (dcm2nii) and imported into the brain imaging analysis software platform, FSL (v5.0.2.1). The FSL automated brain extraction tool (Smith, 2002) was used to remove the skull in the images. A 7-DOF *flirt* (Jenkinson and Smith, 2001; Jenkinson et al., 2002; Greve and Fischl, 2009) linear transformation in FSL was used to obtain registration between the SWI data and mean B_0_ DWI volume. The transformation was necessary because even with image distortion correction due to field inhomogeneity, slight image distortion can still exist. Since these two imaging modalities differ, inter-modal cost functions (correlation ratio or mutual information-based options) were applied depending on which produced the best alignment as assessed visually. Before computation of tractography, the diffusion data was pre-processed using *bedpostx* to estimate the diffusion parameters. The bedpostx function was run with 3 fibers per voxel (*n* = 3) to model crossing fibers. All other parameters were by default: *w* = 1, *b* = 1000, *j* = 1250, *s* = 25, model = monoexponential.

Seed point and waypoint masks, based upon the warped atlas, were defined in the SWI images to extract the following white mater tracts: the medial lemniscus (ML) projecting into the ventralis posterior lateralis pars caudalis (VPLc) nucleus of thalamus, the superior cerebellar peduncle (SCP) projecting into ventralis posterior lateralis pars oralis (VPLo) nucleus of thalamus, and the pallidofugal (PF) tract projecting into ventralis lateralis pars oralis (VLo) and ventralis anterior (VA) nuclei of thalamus (Gallay et al., 2008). To estimate the ML tract, seed points were placed in the ML representation of the caudal pons, and a waypoint was introduced as the entire region of the thalamus anterior to the pulvinar. Similarly, the SCP tract was extracted by placing seed points in the posterior pons, with waypoints at the decussation of SCP, and the entire thalamus. Two subjects (M5 and M7) required an additional seed point in the red nucleus. The PF tract was reconstructed using masks over the entire GPi with a waypoint at the thalamus. These masks were transformed into DWI space using the previously calculated transformation and were used for computing the probabilistic tractography (*probtrackx*, number of samples: 5000, curvature threshold: 0.2, number of steps: 2000). Once completed, the resulting tracts were inversely transformed back into SWI space for 3D visualization using the biomedical computer aided design software, Amira.

### Evaluation of atlas warping and diffusion tensor imaging

Objective evaluation of image registration techniques is non-trivial (Vandenelsen et al., 1993; Wolberg, 1998; Chakravarty et al., 2009), and various groups have reported on methods of evaluation (Dann et al., 1989; Seitz et al., 1990; Evans et al., 1991; Gee et al., 1993; Sorlie et al., 1997; Castro et al., 2006; Chakravarty et al., 2008). In this study, evaluation of the accuracy of our matching technique was achieved through two approaches: (1) electrophysiological recordings within thalamus, and (2) comparison of SWI warped atlas plates to corresponding blockface sections of fixed brain tissue.

#### Electrophysiological mapping

Electrophysiological recordings in the thalamus were performed in subject 4, as described previously (Agnesi et al., 2013). Briefly, a 19-mm cranial window was made over the right hemisphere close to midline keeping the dura intact. A sagittal recording chamber (Crist Instruments) was attached over this cranial window to provide microelectrode access to the ventral nuclei of thalamus. Reconstructed volumes of VPLo and VPLc from the lofted atlas deformation process were imported into a surgical navigation software, Monkey Cicerone (Miocinovic et al., 2007), to guide the electrophysiological mapping of the ventral nuclei of thalamus. A post-operative CT scan was co-registered manually to the MRI in Monkey Cicerone using linear translation and rotation so that microelectrode recording locations could be viewed in the context of reconstructed thalamic nuclei. Single channel tungsten microelectrodes (145–250 μm/diameter) were acutely inserted through the ventral nuclei of thalamus in increments of 10 μm, and electrophysiological spike recordings were performed along each track (*n* = 5 tracks). Neuronal responses to passive manipulation (Agnesi et al., 2013) and low-threshold microstimulation (Vitek et al., 1996) were used to identify regions of VPLo, while responses to tactile brushing of the limbs were used to identify regions of VPLc.

#### Blockface tissue sectioning

At the conclusion of the study, subjects 5 and 6 were deeply anesthetized and euthanized (sodium pentobarbital, 100 mg/kg, i.v.). Transcardial perfusion of room temperature phosphate buffered saline (PBS, pH 7.4) occurred at 50 ml/min for 40 min followed by perfusion of 4% paraformaldehyde in PBS at 4°C at the same rate for 20 min. The brain was post-fixed in PFA for 4 h at 4°C then placed in 15% sucrose in PBS at 4°C for 1–3 days in order to cryoprotect. Sections, which were 50 μm thick, were cut in the coronal direction using a freezing microtome. During sectioning, serial images were acquired from a fixed distance using a Canon EOS Rebel T3i with EF-S 18–55 mm IS II lens. Pitch (dorsal-ventral) and yaw (medial-lateral) angles from the AC-PC line were 17.59° and 4.3° for subject 5 and 6.85° and 1.72° for subject 6. Image resolution was ~62 × 62 μm.

## Results

### Visualization of thalamus using SWI at 7T

#### Hypointensity in the medial and posterior thalamus

**Figure 2:**
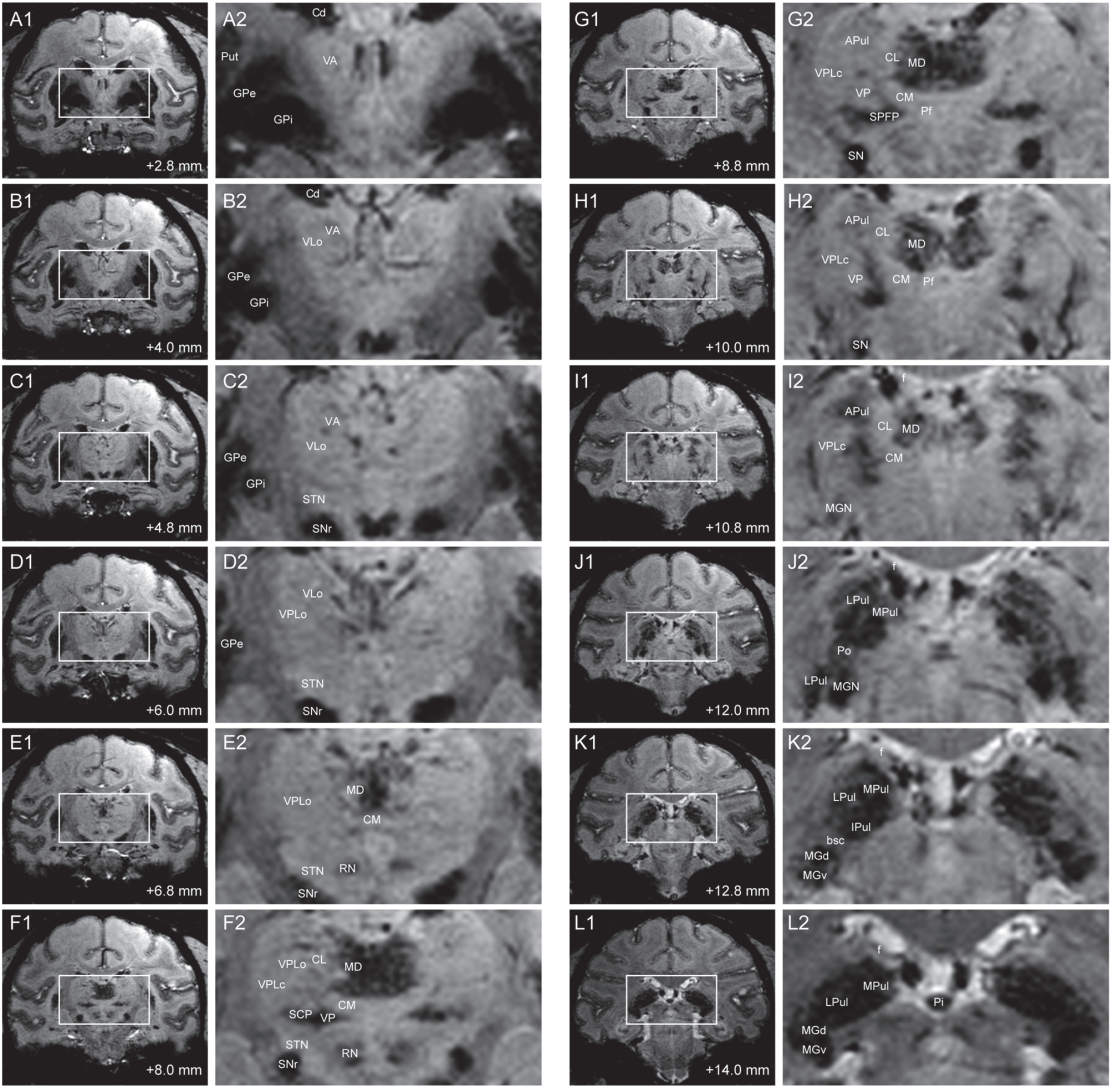
**SWI of coronal slices through thalamus in Subject 2**. Distance demarcations are relative to the midline crossing of the anterior commissure. **(A1–L1)** Coronal SW images. **(A2–L2)** Enlarged images of bounded regions in Panels **(A1–L1)**. (APul: anterior pulvinar, bsc: brachium of the superior colliculus, Cd: caudate nucleus, CL: central lateral nucleus of thalamus, CM: center médian nucleus of thalamus, f: fornix, GPe: globus pallidus externus, GPi: globus pallidus internus, IPul: inferior pulvinar, MGd: dorsal medial geniculate nucleus, MGv: ventral medial geniculate nucleus, MPul: medial pulvinar, LPul: lateral pulvinar, Pf: parafascicular nucleus, Pi: pineal gland, Po: posterior thalamic nuclear group, Put: putamen, RN: red nucleus, SPFP: parvocellular part of the sub-parafascicular nucleus, STN: subthalamic nucleus).

SWI intensity was normalized to the anterior commissure image intensity in each subject so as to mitigate potential variations in MRI scanner sensitivity amongst subjects (see section Age-Dependent Normalized Image Intensity). Image intensity was then compared between and within the thalamic nuclei by superimposing the nonlinearly deformed atlas onto the corresponding susceptibility-weighted images (Figure 2). Using this method, several regions of thalamus, especially in the posterior portion of thalamus, were found to exhibit increased contrast relative to other regions of thalamus and regions external to thalamus. We tested for a significant difference in normalized SWI intensity between anterior and posterior thalamic nuclei. We grouped pixel values from VA and VLo together into one group and those from pulvinar and medial geniculate nucleus (MGN) together into another group (Table 3). We performed two-sample *t*-test between the normalized intensity values between the two groups and found that the difference was significant (*p* = 3.67 × 10^−13^).

Posterior thalamic nuclei including the pulvinar and MGN exhibited hypointensity in the susceptibility-weighted images, which in some subjects showed further demarcations of subregions within each nucleus (Figures 2I–L). The dorsal and ventral aspects of the anterior pulvinar were visible as clustered bands of hypointense regions that extended along the dorsomedial to ventrolateral plane. The anterior pulvinar was bordered by the relatively hyperintense central lateral (CL) nucleus on its medial border, the ventral posterior lateral pars compacta (VPLc) nucleus on its lateral border, and the posterior oralis (PO) nucleus on its ventral border. The PO nucleus was further demarcated by the brachium of the superior colliculus (bsc), which bisects the pulvinar from the MGN (Figure 2K2).

Regions within the mediodorsal nucleus (MD) also exhibited hypointense contrast relative to the center médian nucleus on its ventral border, the central lateral nucleus on its lateral border, and the paraventricular and habenular nuclei on the dorsal border (Figures 2E–I). Additionally, the putative medial (magnocellular) division of the medial dorsal nucleus exhibited greater hypointensity than the lateral division, with the latter exhibiting finger-like projections extending into the central lateral nucleus.

The ventral posterior nucleus, which lies ventrolateral to the center médian nucleus, also exhibited hypointensity that spread medial into the ventral medial nucleus, lateral into the ventral posterior inferior nucleus, and dorsolateral between the center médian nucleus and the VPLc (Figures 2F–H). However, there was relatively little contrast evident between the other ventral nuclei, albeit for a clear demarcation by the relatively hypointense internal capsule on the lateral border and the medial dorsal nucleus on the medial border.

#### Age-dependent normalized image intensity

**Figure 3:**
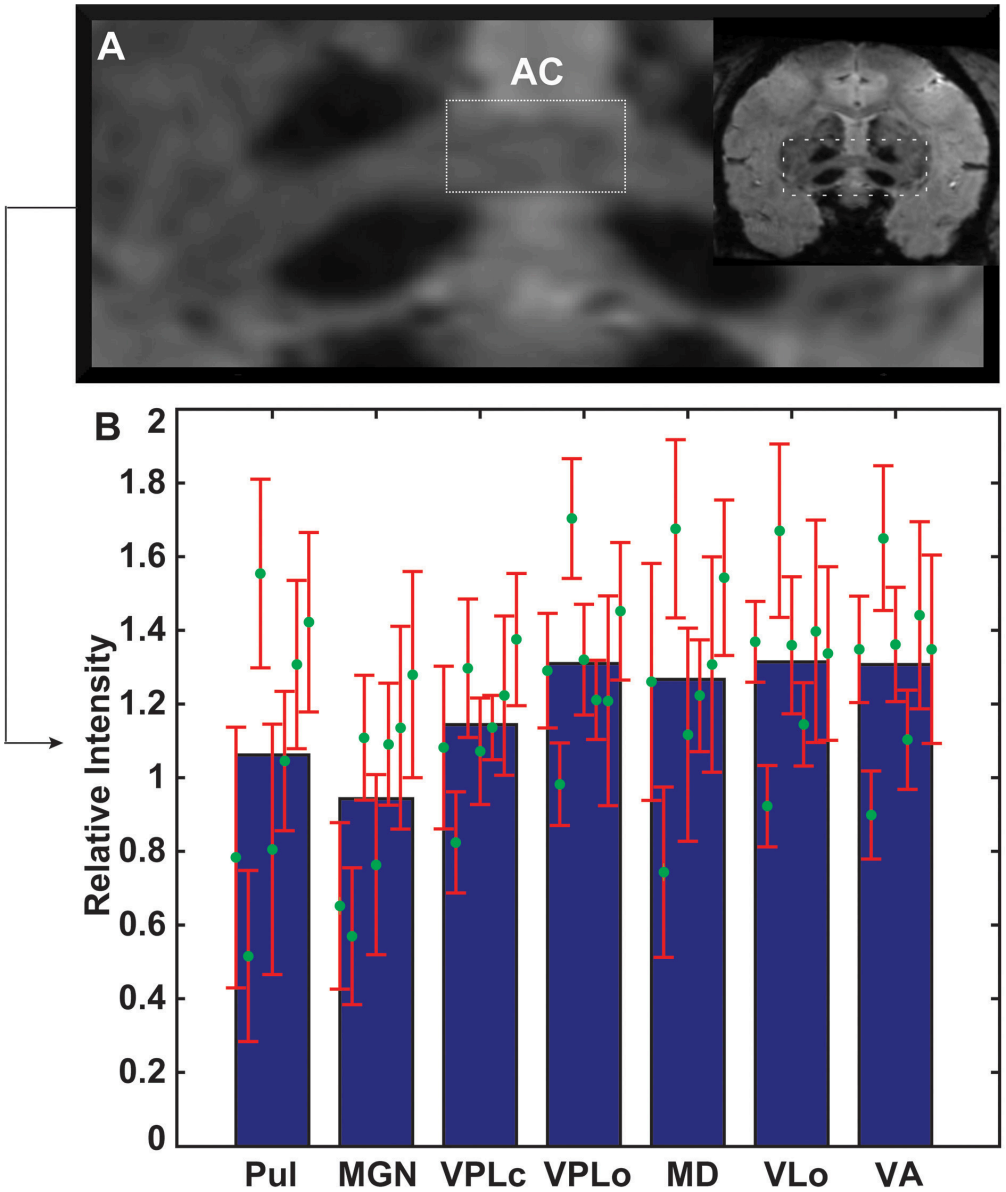
**Age-related changes in thalamic image intensity with SWI. (A)** Anterior commissure in each subject was used to normalize susceptibility-weighted image intensity for each subject**. (B)** Normalized mean intensity of thalamic nuclei (*n* = 7 subjects). Green circles and red error bars mark the mean and standard deviation of normalized intensity for each subject. The mean and standard deviation values for each structure are arranged from left to right from oldest to youngest age.

The contours of pulvinar, medial geniculate nucleus (MGN), VPLc, VPLo, MD, VLo, and VA were segmented and the mean image intensity for each nucleus was calculated by averaging all pixels within relevant contours. The intensity values for seven thalamic nuclei were analyzed and compared amongst all seven subjects, with the images normalized by the subject-specific image intensity of the midline anterior commissure. The anterior commissure tract was chosen for normalization since its intensity did not correlate with age (linear regression analysis, *r*^2^ = 0.0456, slope = 0.4477, *p* = 0.64567). Anterior commissure intensity was calculated from the widest coronal strip of the anterior commissure in each subject (Figure 3A). Several nuclei exhibited trends of increased hypointensity level with age. Correlation analysis (Pearson correlation, df = 5, *p* < 0.05) showed that the normalized mean intensity for nuclei in the posterior half of thalamus had a statistically significant dependence on age (VPLc: *r* = −0.8, Pulvinar: *r* = −0.74, and MGN: *r* = −0.92) (Figure 3B, Table 2). However, this was not the case for the anterior portion of thalamus including the ventral nuclei and MD.

**Table 2:** **Age-related intensity correlations**.

	**VLo**	**MD**	**VPLo**	**VPLc**	**Pulvinar**	**MGN**	**LGN**
*r*	−0.29	−0.59	−0.38	−0.8	−0.74	−0.92	−0.55
*p*	0.5251	0.1677	0.3973	0.0324	0.0484	0.0035	0.1987

Pearson correlation coefficient/p-value (df = 5, p < 0.05).

**Table 3:** **Normalized SWI intensity values**.

	**Subject 1**	**Subject 2**	**Subject 3**	**Subject 4**	**Subject 5**	**Subject 6**	**Subject 7**
Pulvinar	0.78/0.35	0.52/0.23	1.55/0.26	0.81/0.34	1.05/0.19	1.31/0.23	1.42/0.24
MGN	0.65/0.23	0.57/0.19	1.11/0.17	0.76/0.24	1.09/0.17	1.14/0.28	1.28/0.28
VPLc	1.08/0.22	0.82/0.14	1.30/0.19	1.07/0.14	1.14/0.09	1.22/0.22	1.38/0.18
VPLo	1.29/0.16	0.98/0.11	1.70/0.16	1.32/0.15	1.21/0.11	1.21/0.28	1.45/0.19
MD	1.26/0.32	0.74/0.23	1.68/0.24	1.12/0.29	1.22/0.15	1.31/0.29	1.54/0.21
VLo	1.37/0.11	0.92/0.11	1.67/0.24	1.36/0.19	1.14/0.11	1.40/0.30	1.34/0.24
VA	1.34/0.14	0.90/0.12	1.65/0.20	1.36/0.15	1.10/0.13	1.44/0.25	1.35/0.26

(Mean/Standard Deviation).

### Probabilistic tractography of ascending tracts to ventral nuclei in thalamus

**Figure 4:**
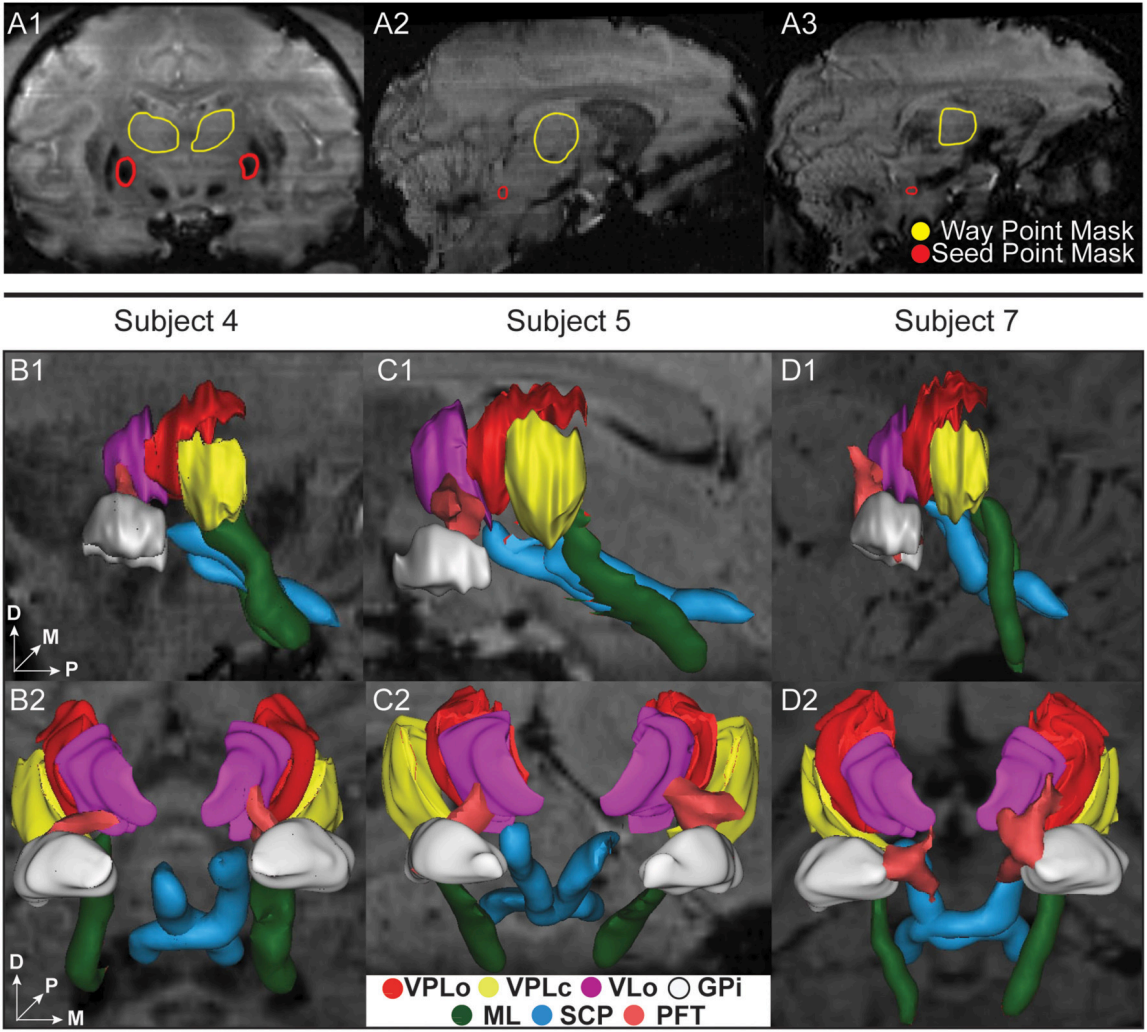
**Ascending fiber tractography to the ventral nuclei of thalamus in three subjects**. **(A1–A3)** Examples of seed point (red) and waypoint (yellow) masks used in the probabilistic tractography. **(A1)** Seed point mask in GPi and waypoint mask in thalamus for reconstruction of the PF tract. **(A2)** Seed point mask of SCP in brainstem and waypoint mask in thalamus for reconstruction of SCP. **(A3)** Seed point mask of ML in brainstem and waypoint mask in thalamus for reconstruction of ML. **(B–D)** Bilateral probabilistic fiber tractography reconstructions for the PF, SCP, and ML tracts and their corresponding thalamic nuclei. The nuclei of the oral (VPLo) and caudal (VPLc) parts of the ventral posterolateral nucleus are reconstructed from series of warped atlas plates. A: anterior, V: ventral, M: medial, D: dorsal, P: posterior.

While most regions of thalamus exhibited contrast sufficient to segment manually or to guide the placement of markers for the nonlinear atlas deformation algorithm, the internal borders of the ventral nuclei were not clearly distinguishable from the SWI. In this case, fiber tractography was used to estimate the ventral nuclei demarcations based upon thalamic afferents coursing along the medial lemniscus (to VPLc), superior cerebellar peduncle (to VPLo), and globus pallidus (to VLo/VA) in three subjects (subjects 4, 5, and 7). The resulting fiber tracts were spatially co-registered to the SWI data and the reconstructed thalamic nuclei. In the case of the ML and PF tracts, the fiber tractography was able to identify the ventral entry point to VPLc and VLo in all three subjects (Figure 4). In the case of the SCP tract, the tractography reconstructions were found to project into or just ventrally adjacent to the VPLo.

### Evaluation of nuclei reconstructions

#### Electrophysiological microelectrode mapping of ventral nuclei in thalamus

**Figure 5:**
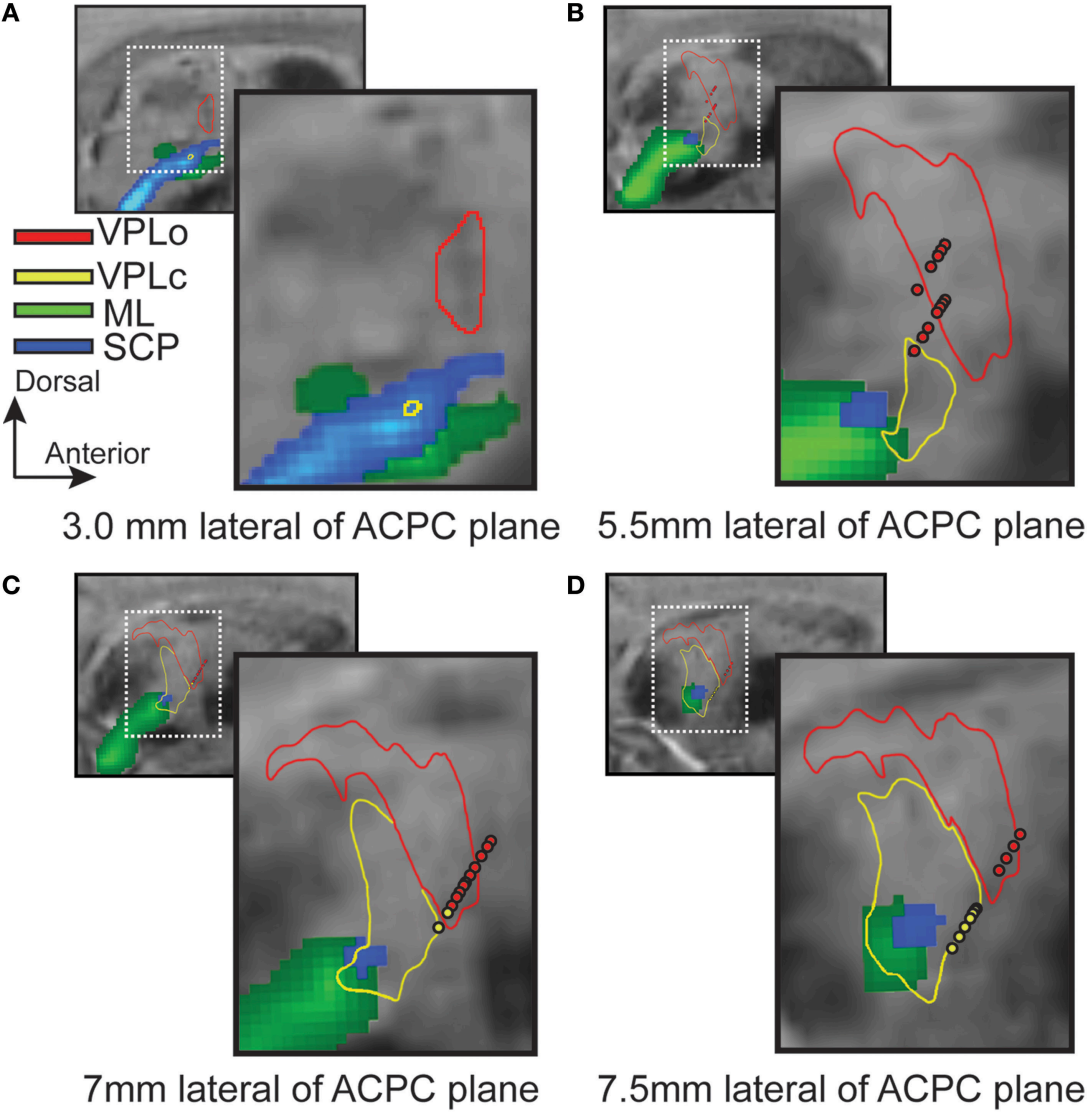
**Superposition of microelectrode recordings with reconstructed thalamic nuclei and probabilistic tractography (subject 4)**. Red and yellow spheres are the locations of VPLo (oral part of the ventral posterolateral nucleus of thalamus) and VPLc (caudal part of the ventral posterolateral nucleus of thalamus) cells, respectively. Red and yellow contours denote the boundaries of VPLo and VPLc, respectively, as defined by the warped brain atlas process. The resultant probabilistic tractography of ML (green) and SCP (blue) are also superimposed on each slice. **(A–D)** Sagittal SW images focused on the posterior thalamic region.

To validate borders between the ventral nuclei, microelectrode spike recordings were performed through a cranial chamber chronically implanted in subject 4. Electrophysiologically identified VPLo and VPLc cells matched closely with the segmented contours and probabilistic tractography predictions across multiple sagittal planes (Figure 5). Small discrepancies at the border regions were observed, possibly due to the spatial spread of the recorded electric fields or slight inaccuracies in the atlas deformation process.

#### Tissue sectioning-based identification of hypointense thalamic nuclei in SWI

**Figure 6:**
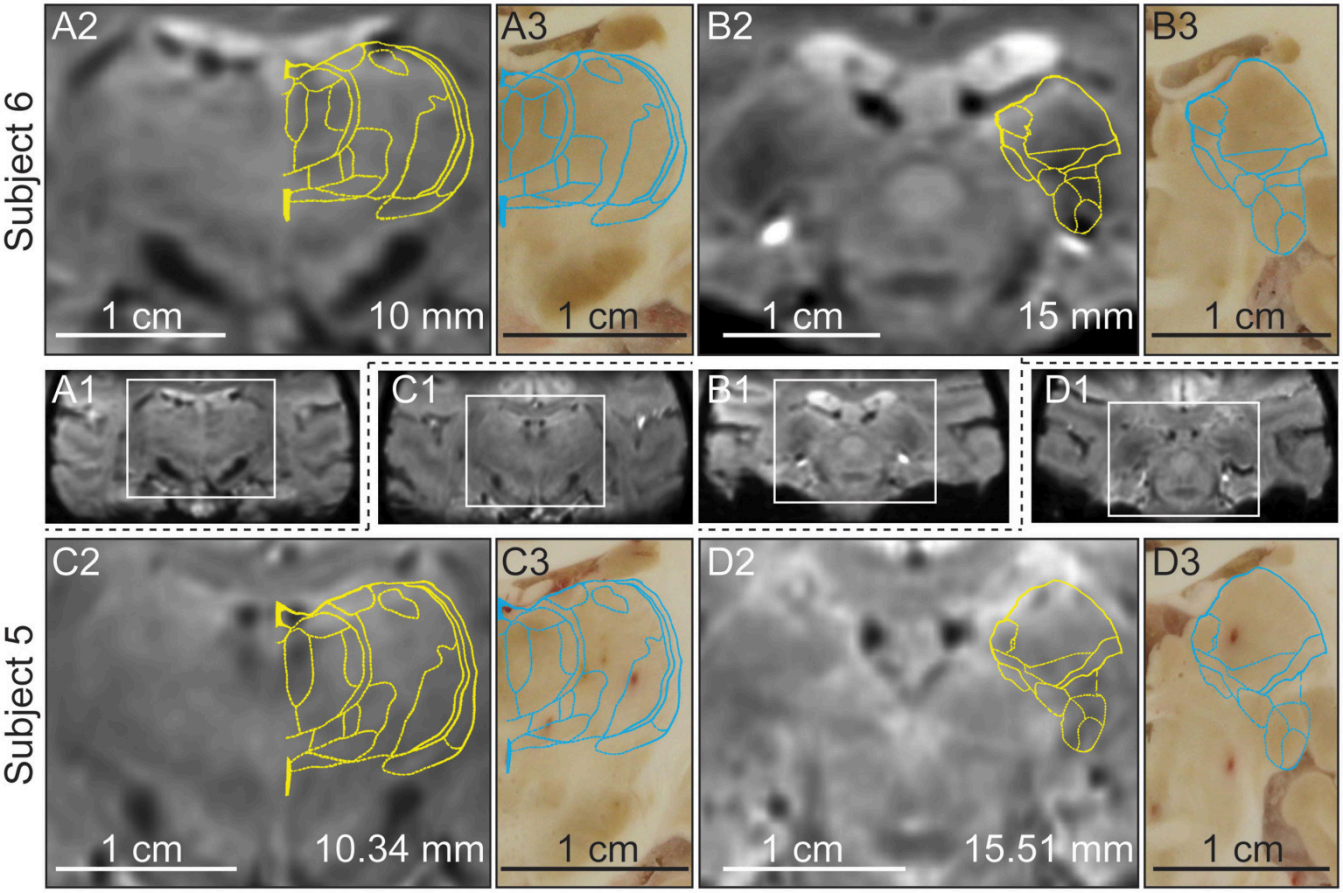
**Relationship between hypointensity in the *in vivo* SWI and *ex vivo* blockface sectioned thalamic nuclei in the same non-human primates (subjects 5 and 6). (A1–D1)** Full brain coronal plane SWI with boxed region enlarged in **(A2–D2)**. **(A2/C2)** SWI of the thalamus in subjects 6 and 5 at 10.34 and 10 mm posterior to AC, respectively. **(A3/C3)** Blockface tissue sections aligned with the images shown in **(A2/C2)**. **(B2/D2)** SWI of the thalamus in subjects 6 and 5 at 15 and 15.51 mm posterior to AC, respectively. **(B3/D3)** Blockface tissue sections aligned with images shown in **(B2/D2)**. The yellow and blue contours show the SWI-deformed atlas plates overlaid on top of the SWI and blockface tissue sections, respectively.

Post-mortem blockface tissue sectioning was performed on subjects 5 and 6 to further validate that the atlas-based warping algorithm results were consistent with anatomical features observed in the *ex vivo* sectioned brain tissue (Figure 6). Two representative sections at the level of the MD/VPLo/VPLc and the Pulvinar/MGN were found in each subject. The MD (Figures 6A2,C2,A3,C3), pulvinar and MGN (Figures 6B2,D2,B3,D3) all appeared hypointense in both the susceptibility-weighted images as well as the tissue section images. Atlas plates were warped to the susceptibility-weighted images and the resulting deformed plates were then linearly scaled (maintaining aspect ratio), slightly rotated (less than 2 ° in either counterclockwise or clockwise directions) and overlaid onto the matching tissue section images. The deformed atlas plates were found to align well with their matching tissue sections, especially in the hypointense pulvinar, MGN, and medial nuclei of the MD (Figures 6A3,B3,C3,D3).

## Discussion

**Figure 7:**
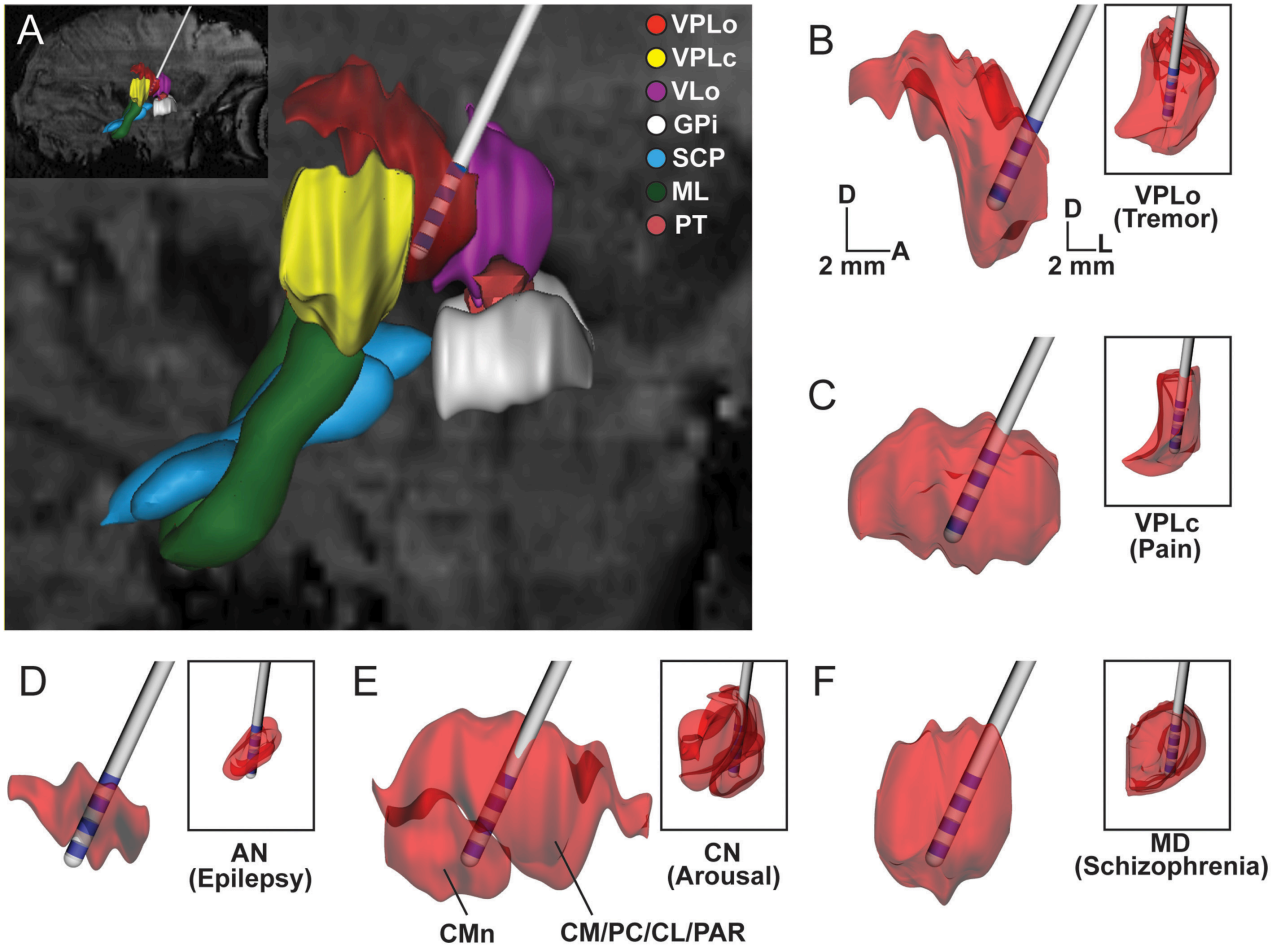
**Subject-specific reconstructions of thalamic nuclei for DBS targeting**. Scaled-down version of the clinical DBS lead shown in the context of thalamic nuclei reconstructed from the susceptibility-weighted images of Subject 4. **(A)** Sagittal view of reconstructed thalamic volumes, afferent fibers, and DBS lead for targeting VPLo to treat tremor disorders. **(B–F)** Large images show sagittal view of thalamic nuclei. Inset shows the coronal view of the same nucleus. Large and small scale bars are for sagittal and coronal images, respectively. D: dorsal, A: anterior, L: lateral.

*In vivo* visualization and demarcation of individual thalamic nuclei is critical for many preclinical and clinical stereotactic neurosurgical procedures targeting thalamus, including implantation of DBS leads (Figure 7). In this study, we show the utility of an *in vivo* multimodal imaging approach using high field 7T SWI and DWI to segment and identify nuclei within the nonhuman primate thalamus (NHP). The results were subsequently validated using electrophysiological recordings and post-mortem tissue sectioning.

### Atlas-based reconstructions

A rich array of 2D or 3D registration methods exist to deform a brain atlas to MR images to identify structures not clearly visible on MR images (Maintz and Viergever, 1998). Most 3D methods work to deform a source surface to fit a target surface by either minimizing difference in distance or energy of the deformation (Bajcsy et al., 1983; Macdonald et al., 1994; Sandor and Leahy, 1994; Cuisenaire et al., 1996; Thompson and Toga, 1996; Davatzikos, 1997; Ganser et al., 2004). This can be achieved by extracting ventricular and cortical surfaces from MR images and matching them to corresponding surfaces on a 3D atlas. The computed deformation based on matching these surfaces is then propagated throughout the entire volume. Although such methods may be appropriate for matching cortical surfaces, they essentially relegate the deformation of deep structures such as the thalamus to be a by-product of cortical and ventricular deformation (Sandor and Leahy, 1994).

One 2D solution well-suited for atlas-based localization of structures lacking contrast in MR images involves the MLS method embodying the idea of “as rigid as possible” image deformation (Alexa et al., 2000; Igarashi et al., 2005) which minimizes the amount of local scaling and shearing once certain constraints are satisfied. This more conservative approach incorporates the most reliable anatomical information contained in the MR for the deformation process, and does not require defining features of an image and guessing for model parameters (Bajcsy et al., 1983; Gee et al., 1993). Instead, the process requires setting a set of identical control points between the atlas and MR image to guide the deformation process. When coupled with high field imaging approaches with higher spatial resolution (Lenglet et al., 2012), the user can simply crop out all but the region of interest (e.g., thalamus) and perform a very local deformation. This approach is computationally efficient on top of an already fast and easy-to-implement algorithm. The result of the deformation is smooth and more realistic than the popular thin-plate spline approach (Bookstein, 1989; Schaefer et al., 2006). For these reasons we implemented the MLS method to take advantage of the increased contrast within thalamus at 7T to help further demarcate borders between thalamic nuclei.

### Susceptibility-weighted imaging of thalamus

Analysis of the SWI data across the seven subjects showed: (1) relative hypointensity in internal capsule compared with the ventral nuclei of thalamus, (2) thalamic regions of hypointensity most notably in the posterior half of thalamus and in the medial dorsal nucleus, and (3) positive correlations of image hypointensity in the posterior half of thalamus with age. SWI is sensitive to a difference in magnetic susceptibility in tissues and can be used to measure iron content (Haacke et al., 2009b). Regions with higher iron content exhibit larger, linearly correlated phase shifts and will appear hypointense in the SWI data (Ogg et al., 1999; Hopp et al., 2010). Iron in the form of ferritin and hemosiderin is stored in oligodendrocytes (Francois et al., 1981; Dwork et al., 1988; Levine and Macklin, 1990; Connor and Menzies, 1996; Schenck and Zimmerman, 2004) and has been found in relatively rich deposits in certain brain regions (Haacke et al., 2009a). Consistent with our results in the posterior half of thalamus, brain iron has been shown to accumulate with age (Hallgren and Sourander, 1958), and an elevation in iron concentration in certain regions is known to occur in neurodegenerative diseases, including for example the substantia nigra and globus pallidus in Parkinson's disease (Dexter et al., 1991; Chen et al., 1993; Griffiths and Crossman, 1993; Loeffler et al., 1995) and hippocampus in Alzheimer's disease and Parkinson's disease (Small et al., 1999, 2000). In order to detect potential outliers in our data, we calculated Cook's distance (Cook, 1977) for each nuclei using the age of the seven subjects as the independent variable and the average normalized SWI intensity values as the explanatory variable. A data point is considered an outlier if its Cook's distance value exceeded three times the average Cook's distance across all seven subjects (for a given nucleus). This analysis indicated that subject two is an outlier in all nuclei except pulvinar and MGN. These two posterior also demonstrated statistical significance in correlation between normalized SWI intensity and age. It should also be noted that SWI is not exclusive in its ability to demarcate nuclei within thalamus, and other approaches including low-field T1 and T2-weighted imaging have been used (Deoni et al., 2005).

### Cross-validation of the computationally segmented thalamic nuclei derived from SWI

In this study, we applied multiple tools to cross-validate the segmentation of thalamic nuclei based on 7T SWI data including probabilistic fiber tractography, electrophysiology, and *ex vivo* blockface tissue sectioning. This multi-modal approach was feasible given the animal model preparation used.

#### Demarcation of the ventral nuclei using probabilistic tractography

Previous studies have found considerable variability in the location of generic atlas-based target points in thalamic nuclei in relation to major neighboring fiber tracts in individual patients, suggesting the need for individualized methods that can target structures not directly visible on conventional MRI (Anthofer et al., 2014). One approach to subject-specific mapping of thalamic nuclei includes probabilistic fiber tractography for reconstructing white matter pathways (Zakszewski et al., 2014) into the thalamus including those originating from globus pallidus internus (PF tracts) (Lenglet et al., 2012) and cortex (Behrens et al., 2003). Here, we extend these studies showing nearly complete demarcation of the ventral nuclei utilizing ascending ML, SCP, and PF fiber tracts. This approach provided important data to verify the atlas plate to SWI slice alignment for the anterior portion of the thalamus. The trajectories of the fiber tracts projecting into the subject-specific ventral nuclei reconstructions (i.e., VPLc, VPLo, and VLo/VA) were consistent across the three subjects.

#### Electrophysiological cross-validation

The accuracy of the warping process was also verified by *in vivo* electrophysiological recording in the ventral thalamic nuclei. Cells were categorized based on their responsiveness to proprioceptive and microstimulation excitable (VPLo) and tactile (VPLc) input. While the locations of these cell types aligned well with both the deformed atlas and fiber tractography results, there were small discrepancies at the border between the nuclei. In this case, additional deformation methods can be applied to further reduce these small discrepancies (Lujan et al., 2009).

#### Blockface tissue sectioning cross-validation

To avoid deformation of the tissue during histological processing, we chose to take blockface photographs of the brain during sectioning. Two types of deformations may still occur during the preparation of brain sections: 3D deformation caused by extraction of the brain from the skull and 2D deformation caused by the sectioning process (Dauguet et al., 2007). The three-dimensional deformation stems from loss of cerebrospinal fluid and blood and subsequent mechanical effects from gravity. Two-dimensional deformation results from shearing and tearing during cutting of the brain tissue and shrinkage due to changes in tissue temperature and hydration. Natural shade differences of frozen brain tissue were found to be sufficient for identifying many of the major thalamic nuclei. Based on this analysis, the relative positions of the thalamic nuclei on the blockface photographs resulted in consistent registration, where only linear scaling (no change in aspect ratio) and slight rotations (less than 1°) were needed when overlaying the SWI-warped atlas plates onto the blockface sections. This registration was most visible for MD, pulvinar, and MGN borders with their respective hypointense regions in the MR images. In addition, the borders of the thalamus in the medial/lateral and dorsal/ventral directions also aligned well. The tissue sectioning validation demonstrated that the image warping approach could in the future utilize contrast not only between gray/white matter boundaries, but also between different thalamic nuclei to guide accurate segmentation of nuclei within thalamus.

### Limitations

There are several points to consider in the interpretation of the results. First, the data set included six females and only one young male rhesus macaque. These subjects were selected in part because their cranial musculature was minimal allowing for the receiver coils to be placed closer to the brain (Zitella et al., 2015). Another limitation is the use of an atlas that is particular to one NHP, along with its own nomenclature and criteria of demarcation (Macchi and Jones, 1997). However, the approach itself is one that can be extended to other brain atlases based on cytoarchitectonic features. While the fiber tractography and warping methodology results aligned reasonably well with the histological blockface images and electrophysiological results, there was some degree of misalignment especially in the caudate and substantia nigra regions with the histological images. This registration error likely stemmed in part from nonlinear deformations that occurred as part of the perfusion, fixation, and freezing processes. Further, the histological coronal sections were sliced at a slight pitch and yaw from the AC-PC line. Future studies that utilize 3D rendering of histology-based fiber tracts would be useful to further validate tractography and atlas warping methods. Lastly, we are limited by the relatively small sample size in the number of subjects with 7T SWI data. The analysis of correlation between normalized SWI intensity and subject age would benefit from a larger sample size. However, we are confident based on the outlier detection analysis that the posterior nuclei show significant correlation between average SWI intensity and age.

## Application to DBS targeting

The multimodal imaging approaches shown here provided enhanced visualization of thalamic nuclei, which can be critical for preclinical and clinical stereotactic neurosurgery procedures (Abosch et al., 2010) (Figure 7). Defining thalamic nuclei through non-invasive means is especially important given that most nuclei have been targets for deep brain stimulation therapies and the precise locations, shapes, and sizes of these nuclei vary amongst subjects. In this way, the combined use of imaging techniques described in this study can assist in neurosurgical navigation of DBS targets in a given subject (Kamiryo and Laws, 1996; Dormont et al., 1997; Dipierro et al., 1999). Additionally, the segmented nuclei reconstructions can also aid in the development of more accurate computational models of DBS (Mcintyre et al., 2004; Kuncel et al., 2008; Keane et al., 2012; Zitella et al., 2013) to retrospectively quantify the neural pathways modulated by thalamic DBS therapy (Xiao and Johnson, 2015) or prospectively predict the stimulation settings necessary to target those pathways on a subject-specific basis (Xiao et al., 2016).

## Author contributions

YX, GA, EY, NH, and MJ designed research; all authors performed the research; YX, LZ, YD, BT, DK, and MJ analyzed the data; YX and MJ wrote the paper.

### Conflict of interest statement

The authors declare that the research was conducted in the absence of any commercial or financial relationships that could be construed as a potential conflict of interest.
